# Supplementary material for: Genetic Diversity and Selection Signal Analysis of Hu Sheep Based on SNP50K BeadChip
Source: Animals (Basel). 2024 Sep 26;14(19):2784. doi: 10.3390/ani14192784 (PMC11476051; doi:10.3390/ani14192784)
Supplement: Supplementary file 1 [file animals-14-02784-s001.zip › Supplementary Figure S1-S2.pdf]

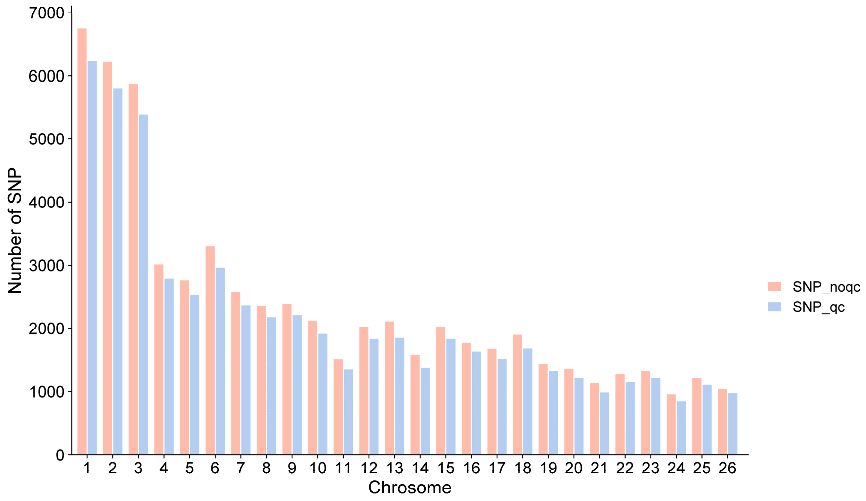

Figure S1. The distribution of SNPs on each chromosome before and after quality control.

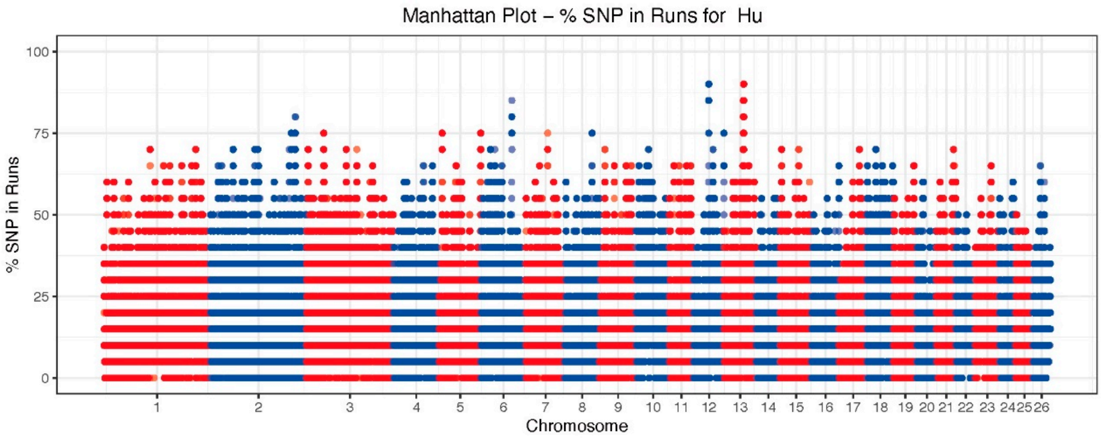

Figure S2. Manhattan plot of the occurrences (%) of a SNP in ROHs across Hu individuals.
